# Selenium Level and Dyslipidemia in Rural Elderly Chinese

**DOI:** 10.1371/journal.pone.0136706

**Published:** 2015-09-18

**Authors:** Liqin Su, Sujuan Gao, Frederick W. Unverzagt, Yibin Cheng, Ann M. Hake, Pengju Xin, Chen Chen, Jingyi Liu, Feng Ma, Jianchao Bian, Ping Li, Yinlong Jin

**Affiliations:** 1 Department of Environmental Impact Assessment, Institute for Environmental Health and Related Product Safety, Chinese Center for Disease Control and Prevention, Beijing, China; 2 Department of Biostatistics, Indiana University School of Medicine, Indianapolis, Indiana, United States of America; 3 Department of Psychiatry, Indiana University School of Medicine, Indianapolis, Indiana, United States of America; 4 Department of Environmental Epidemiology, Institute for Environmental Health and Related Product Safety, Chinese Center for Disease Control and Prevention, Beijing, China; 5 Department of Neurology, Indiana University School of Medicine, Indianapolis, Indiana, United States of America; 6 Shandong Institute for Prevention and Treatment of Endemic Disease in China, Jinan, China; 7 Sichuan Provincial Center for Disease Control and Prevention in China, Chengdu, China; Robert Gordon University, UNITED KINGDOM

## Abstract

**Objective:**

Higher selenium level has been hypothesized to have the potential to reduce the risk of cardiovascular diseases including dyslipidemia. However, results from previous studies are inconsistent. This study aims to determine the association between selenium level and dyslipidemia in elderly Chinese with relatively low selenium status.

**Methods:**

A cross-sectional study of 1859 participants aged 65 or older from four rural counties in China was conducted. Serum total cholesterol (TC), triglycerides (TG), high density lipoprotein-cholesterol (HDLC) and low-density lipoprotein-cholesterol (LDLC), nail selenium concentration and *APOE* genotype were measured in all subjects. The four types of dyslipidemia were defined as >5.17mmol/L for High-TC, >1.69 mmol/L for High-TG, >3.36 mmol/L for High-LDLC, and <1.04 mmol/L for Low-HDLC according to Chinese Guidelines on Prevention and Treatment of Dyslipidemia in Adults. Logistic models adjusting for age, gender, *APOE* genotype, body mass index, alcohol consumption, smoking, physical activity, medication use for cardiovascular diseases were used to examine the relationship between selenium levels and the risk of dyslipidemia.

**Results:**

Mean nail selenium concentration was 0.465*μ*g/gin this sample. Rates for High-TC, High-LDLC, High-TG, Low-HDLC were 18.13%, 13.23%, 12.21% and 32.76% respectively. Results from logistic models indicated that higher selenium levels were significantly associated with higher risk of High-TC, High-LDLC and lower risk of Low-HDLC adjusting for covariates (p < 0.0001). Compared with the lowest selenium quartile group, participants in selenium quartile groups 2, 3 and 4 had significantly higher rates of High-TC, High-LDLC, High-TG, and lower rate of Low-HDLC adjusting for covariates. No significant association was observed between selenium level and the risk of High-TG. *APOEε*4 carriers had higher rates of High-TC and High-LDLC. There was no interaction between selenium level and APOE with the rates of dyslipidemia.

**Conclusions:**

Our results suggest long-term selenium exposure level may be associated with the risk of dyslipidemia in elderly population. Future studies are needed to examine the underlying mechanism of the association.

## Introduction

Selenium (Se) is an essential micronutrient with antioxidant properties and it has been hypothesized to have the potential to prevent cardiovascular disease (CVD) and other chronic diseases [1,2]. Experimental studies suggest that selenium supplement may reduce the risk of CVD [3–7]. However, in population based studies, the relationship between selenium levels and the prevalence of CVD was inconsistent [8–11]. Recently, more concern has been raised about possible adverse cardio-metabolic effects of high selenium status on CVD, such as increased risk of hyperlipidemia [12], which is one of the pathogenetic bases of cardiovascular diseases.

As a key component of selenoproteins, selenium plays important roles against oxidative stress, which is also important to lipid metabolism [13]. Animal studies had indicated that selenium deficiency may modulate lipoprotein metabolism [14–16]. Selenium supplementation has also been reported to reduce non-HDL cholesterol in a randomized trial of elderly volunteers [17]. However, results from epidemiological studies have found that higher selenium levels were associated with higher lipid levels [18–22]. Most of these studies were from developed countries where selenium supplements were common and individuals with high cholesterol levels were generally treated with lipid lowering medications. Thus the positive association between selenium and lipid could have resulted from reverse causation that individuals with hyperlipidemia were more likely to take dietary supplements including selenium.

The rural elderly Chinese population represents a unique opportunity for studying the relationship between long-term selenium exposure and the risk of hyperlipidemia. The rural Chinese are stable, with most living in the same village throughout their entire life. They also consume food that is locally grown and rarely take dietary supplements. In preliminary analysis using baseline data of Selenium and Cognitive Decline Study Cohort with a relatively small sample [23], we found a linkage between selenium exposure level and serum lipid levels [24]. In addition, we reported that carriers of the *APOE* ε4 allele had significantly lower selenium levels measured in nail samples than non-carriers after adjusting for other significant covariates and controlling for estimated dietary selenium intake [25]. In this paper, we report our findings from a large cross-sectional study on the association between selenium levels and the risk of dyslipidemia controlling for *APOE* genotype in this elderly Chinese sample.

## Materials and Methods

### Study Population

Participants(n = 1859) aged 65 or older from four counties in China were included in this study: 1067 participants were from the original Selenium and Cognitive Decline Study Cohort enrolled between 2003 and 2005 as described previously [25], and 792 participants were enrolled between 2010 and 2012. Two sites were from Sichuan Province in southwestern China, and the other two sites were from Shandong Province in eastern China. The two provinces were selected because each province can identify two sites with differing selenium levels with similar levels on other trace element levels according to previous study results by a Chinese research group [26].

### Ethics Statement

The study was approved by the Indiana University Institutional Review Board and the Institute for Environmental Health and Related Safety, Chinese Center for Disease Control and Prevention. All participants provided their written informed consent prior to participating in the study.

### Selenium Measures

Fingernail samples were collected and stored in clean plastic bags at the time of interview. Fingernail samples were firstly cleaned by ultrasound followed by socking in nitric acid and perchloric acid and digested on electric hot plate, then reduced by hydrochloric acid. The pretreated samples were restored to room temperature, followed by adding potassium ferricyanide and hydrochloric acid, and diluted with deionized water to volume (10 mL). The concentration of selenium was determined by atomic fluorescence spectrometer at the wavelength of 196.0 nm.

### Serum Lipid Measures

Fasting peripheral blood samples were collected and serum was separated within four hours during the 2010–2012 survey. All samples were stored in -80°C refrigerator before analysis. Serum total cholesterol (TC), triglycerides (TG), high density lipoprotein-cholesterol (HDLC) and low-density lipoprotein-cholesterol (LDLC) were measured using the Roche Diagnostic Kits by Hitachi Automatic Biochemistry Analyzer 9700. The definitions for four types of dyslipidemia were >5.17mmol/L for High-TC, >1.69 mmol/L for High-TG, >3.36 mmol/L for High-LDLC, and <1.04 mmol/L for Low-HDLC according to Chinese Guidelines on Prevention and Treatment of Dyslipidemia in Adults [27].

### *APOE* Genotyping

Genomic DNA was extracted with Bio Teke Whole Blood DNA Extraction Kits following the manufacturer’s instructions. Two single-nucleotide polymorphisms (SNPs) at triplet 112 (rs429385) and 158 (rs7412) were determined by the multiplex tetra-primer amplification refractory mutation system (T-ARMS) PCR reactions according to literature [28]. Participants were classified into two groups, *APOEε*4 carriers (*ε*2/*ε*4, *ε3*/*ε*4, *ε*4/*ε*4) and non-carriers (*ε*2/*ε*2, *ε3*/*ε*3, *ε*2/*ε*3) in the analysis.

### Collection of Other Risk Factors

Information on age, gender ethnicity, alcohol consumption, smoking history, medication use for cardiovascular diseases, and physical activity were collected by means of a questionnaire. All participants in this study are ethnically Han Chinese. Physical activity was classified into three categories of low, moderate or high according to the Guidelines for Data Processing and Analysis of the International Physical Activity Questionnaire (IPAQ) [29]. Height and weight were measured during interview. Body mass index (BMI) was calculated as weight in kilogram divided by height in square meters.

### Statistical Analysis

Descriptive data are expressed as means and standard deviation, percentages or percentiles. To better capture the association between selenium levels and lipid biomarker levels, covariates including age, gender, *APOEε*4 genotype, BMI, alcohol consumption, smoking, medication use for cardiovascular diseases, and physical activity were considered in the analysis. Participants were divided into four groups according to quartiles of nail selenium concentration as Q1, Q2, Q3 and Q4. The cut off values for selenium were 0.320, 0.467 and 0.568 *μ*g/g.

Analysis of variance, the Kruskal-Wallis test, or chi-squared test was used for univariate analysis. Unconditional logistic regression models with 0.05 as significance level were used for multivariate analysis. Separate logistic regression model was used to estimate odds ratios and 95% confidence intervals for the four types of dyslipidemia. We used two models with progressive degrees of adjustment. Model 1 adjusted for age, gender and *APOEε*4 genotype. Model 2 additionally adjusted for BMI, smoking, alcohol consumption, physical activity and medication use for cardiovascular diseases. Potential interaction between *APOEε*4 genotype and selenium level for the risk of dyslipidemia was also examined in separate models. All analyses were performed using SAS9.1 for Windows (SAS Institute Inc., Cary, North Carolina, USA). *P*<0.05 was considered statistically significant.

## Results

Characteristics of the overall population and in the four groups defined by nail selenium quartile were shown in Table 1. Mean selenium concentration in the overall population was 0.465*μ*g/g with ranges from 0.078 to 4.217 *μ*g/g. Mean selenium concentration of each quartile group was 0.232, 0.408, 0.516 and 0.705 *μ*g/g, respectively. No difference in age or *APOEε*4 genotype was observed among the four selenium quartile groups, while significant differences in the remaining variables were observed.

**Table 1:** Characteristics of participants by nail selenium quartile groups.

	Quartile groups of nail selenium level					
Characteristics*	Q1(n = 466)	Q2 (n = 468)	Q3 (n = 462)	Q4 (n = 463)	Total (n = 1859)	*P* Value
Nail selenium (*μ*g/g)	0.232±0.054	0.408±0.040	0.516±0.029	0.705±0.268	0.465±0.221	
Age (years)	73.2±6.0	73.6±6.0	74.1±6.0	74.2±5.7	73.8±5.9	0.0573
Female (%)	43.78	47.86	57.36	65.23	53.52	<0.0001
BMI (kg/m^2^)	21.75±3.05	22.55±3.74	23.53±3.74	24.53±3.94	23.09±3.78	<0.0001
Alcohol consumer (%)	48.28	41.45	33.12	31.53	38.62	<0.0001
Smoker (%)	39.27	47.65	39.39	32.18	39.64	<0.0001
Medication use (%)	15.24	30.34	36.15	46.44	32.01	<0.0001
*APOE ε*4 carriers (%)	13.73	18.80	15.80	15.12	15.87	0.1865
Physical activity						0.0086
Low (%)	2.58	4.91	5.21	6.26	4.74	
Moderate (%)	13.98	14.74	19.52	16.85	16.26	
High (%)	83.44	80.34	75.27	76.89	79.00	

* Characteristics were described as mean ± SD or percentages.

In the overall population, rates of High-TC, High-LDLC, High-TG and Low-HDLC were 18.13%, 13.23%, 12.21% and 32.76%, respectively, as shown in Table 2. Rates of High-TC, High-LDLC, High-TG were significantly higher in Q2, Q3 and Q4 groups compared to the Q1 group (p<0.0001), while rate of Low-HDLC in the Q2, Q3 and Q4 groups were significantly lower (p<0.0001). Compared with *APOEε*4 non-carriers, rates of High-TC and High-LDLC in *APOEε*4 carriers were higher (p<0.0001). Multivariable-adjusted odds ratios of *APOEε*4 carriers were 1.97(95%CI: 1.46, 2.66) for High-TC and 1.66(95%CI: 1.17, 2.35) for High-LDLC, respectively. However, no interaction between *APOEε*4 and selenium level was observed on the risk of dyslipidemia.

**Table 2:** Rates of four types of dyslipidemia by *APOE* genotype and by nail selenium quartile groups.

Group	N	Mean nail selenium (*μ*g/g)	High-TC (%)	High-LDLC (%)	High-TG (%)	Low-HDLC (%)
Total	1859	0.465±0.221	18.13	13.23	12.21	32.76
Selenium Quartiles (p-value)			<0.0001	<0.0001	<0.0001	<0.0001
Q1 group	466	0.232±0.054	4.94	2.36	6.44	44.21
Q2 group	468	0.408±0.040	20.30	13.25	16.03	30.34
Q3 group	462	0.516±0.029	26.84	18.83	15.80	26.19
Q4 group	463	0.705±0.268	20.52	18.57	10.58	30.24
*APOE ε*4 status (p-value)			<0.0001	0.0089	0.3345	0.8244
*APOE ε*4 carrier	295	0.465±0.193	27.46	17.97	13.90	32.20
*APOEε*4 non-carrier	1564	0.465±0.225	16.37	12.34	11.89	32.86

Separate logistic regression analyses indicated that selenium level was significantly associated with High-TC, High-LDLC and Low-HDLC. Multivariable-adjusted odds ratios for four types of dyslipidemia with the lowest selenium quartile group(Q1)as the reference group were calculated using separate logistic models, shown in Table 3. Results indicated that participants in groups Q2, Q3 and Q4 had higher risk of High-TC and High-LDLC, while participants in these groups had lower risk of Low-HDLC compared with the Q1 group after adjusting for other risk factors. Since the confidence intervals for selenium quartile groups Q2, Q3 and Q4 overlap, there were no significant differences in rates of dyslipidemia among these three groups of individuals. No statistically significant differences were found among the four selenium quartile groups for High-TG.

**Table 3:** Multivariable-adjusted odds ratios for four types of dyslipidemia by nail selenium quartile groups.

Dyslipidemia	Selenium Q1 group	Selenium Q2 group		Selenium Q3 group		Selenium Q4 group		*P* trend
	Reference	OR	95%CI	OR	95%CI	OR	95%CI	
High-TC								
Model 1	1.00	4.70	(2.91,7.59)	6.52	(4.07,10.44)	4.31	(2.67,6.98)	<0.0001
Model 2	1.00	4.51	(2.78,7.30)	6.13	(3.80,9.88)	4.02	(2.45,6.58)	<0.0001
High-LDLC								
Model 1	1.00	6.03	(3.13,11.65)	8.67	(4.55,16.52)	8.04	(4.21,15.34)	<0.0001
Model 2	1.00	5.49	(2.83,10.63)	7.55	(3.93,14.48)	6.48	(3.35,12.51)	<0.0001
High-TG								
Model 1	1.00	2.76	(1.76,4.32)	2.51	(1.60,3.94)	1.49	(0.92,2.41)	0.3232
Model 2	1.00	2.48	(1.58,3.92)	2.05	(1.29,3.26)	1.12	(0.68,1.85)	0.4956
Low-HDLC								
Model 1	1.00	0.55	(0.42,0.72)	0.45	(0.34,0.60)	0.56	(0.42,0.73)	<0.0001
Model 2	1.00	0.48	(0.36,0.64)	0.35	(0.26,0.46)	0.38	(0.28,0.51)	<0.0001

Model 1: Logistic regression model adjusted for age, gender and *APOEε*4 genotype. Age was classified into two groups, the cutoff value was 75. The reported parameter estimates were odds ratios. Model 2: Additionally adjusted for BMI, smoking, alcohol consumption, physical activity and medication use for cardiovascular diseases. BMI was classified into three groups, the cutoff values were 18.5 and 25.

## Discussion

In this large cross-sectional study in elderly Chinese over the age of 65, we found that selenium level was associated with dyslipidemia. In particular, participants in higher selenium quartile groups had higher rates of High-TC, High-LDLC, and lower rate of Low-HDLC compared to those in the lowest selenium quartile.

Our findings are consistent with published results from other population based studies evaluating the relationship between selenium status and the risk of dyslipidemia. A recent case-control study focusing on the end-point of dyslipidemia found similar results as our study that selenium levels measured in hair samples were significantly higher in patients with hyperlipidemia than those with normal lipid levels [20]. Many studies have focused on the relationship between blood selenium level and lipid profiles. In selenium-replete populations, positive associations of serum selenium with TC, LDLC and TG concentrations were observed [8, 9, 30]. However, the associations were inconsistent in populations with low serum selenium concentration [31–36]. A cross-sectional study in the 2000–2001 UK National Diet and Nutrition Survey suggested that higher plasma selenium was associated with increased total and non-HDL cholesterol levels in the UK adult population with lower selenium status [19]. A cohort study in Finland where selenium levels were among the lowest in the world until the early 1980s found a positive cross-sectional association between serum selenium status and serum lipids [21]. However, longitudinal analysis in the same cohort did not support the causality of this link. Our results, also cross-sectional in nature, will need to be confirmed in future longitudinal studies.

There has been one randomized trial in elderly volunteers reporting that selenium supplementation resulted in modest reduction in non-HDL cholesterol over a 6-month intervention period [17]. Interestingly, in the cross-sectional analyses at baseline reported in the RCT trial, higher plasma selenium was associated with higher total and HDL cholesterol, similar to our results. However, in longitudinal analyses of this RCT, increasing plasma selenium concentrations from baseline to 6 months were associated with decreasing total cholesterol levels, non-HDL cholesterol levels, and with increasing HDL cholesterol levels. A potential explanation for the cross-sectional relationship between higher selenium and higher lipid levels is a shared enzyme, 3-hydroxy-3-methylglutaryl coenzyme A reductase, which acts through the mevalonate pathway that affects both selenium and lipids [37]. It has been shown that patients treated with statins had lower plasma selenium concentrations than patients who did not receive treatment [38]. These results also highlight the necessity for more studies utilizing longitudinal data.

There were also selenium supplementation trials with cardiovascular disease (CVD) as the outcome. However, the two largest trials that were conducted in the USA (SELECT and NPC) found no statistically significant effects of selenium supplementation on CVD events, CVD mortality or all-cause mortality [39–41]. And it is not clear whether lipid levels were different between the selenium supplement group and the placebo group.

The mechanisms underlying the association between high selenium exposure and lipid metabolism are not clear, although there are studies suggesting a potential pathway through oxidative stress. A recent study reported a nonlinear dose-response relationship between selenium exposure and oxidative stress biomarkers suggesting that higher selenium levels increased oxidative stress [42]. In addition, there were also findings indicating that selenium can be identified in human lipoproteins [43] and experimental studies suggesting that selenium may play an important role in lipid peroxidation and lipoprotein metabolism [14–16,44].

In this study, we found that participants in the top three selenium quartiles had increased rates of dyslipidemia and the three groups did not differ significantly in rates of dyslipidemia as indicated by the overlapping confidence intervals for the odds ratio estimates. Our results seem to point to a threshold effect of selenium levels at the second quartile. However, an inverse U-shape relationship could also exist between selenium level and dyslipidemia [45], but we will not be able to detect the downward trend at the very high selenium level from data in this cohort.

In our study, we also observed that *APOEε*4 carrier was a significant risk factor for High-TC and High-LDLC, indicating that genetic factors have a direct effect on cholesterol metabolism. Recent studies on the association between *APOE* polymorphism and risk of cardiovascular disease had reached similar conclusions [46–49], and more evidence could be found in studies on *APOE* polymorphism and lipid levels [50–53]. On the question of potential interactions between *APOE* genotype and selenium level for the development of dyslipidemia, animal studies demonstrated that selenium supplementation was responsible for down regulation of apoB expression during hypercholesterolemia [54, 55], and could increase the LDL-receptor activity [56,57]. Analysis of plasma proteins in Trsp knockout mouse revealed increases in apolipoprotein E level accompanied with elevated plasma cholesterol levels, providing the first evidence that selenoproteins may play a role in regulating lipoprotein biosynthesis and metabolism [58]. Although associations between selenium level and *APOE* genotype were found in population based studies [25, 59], we found an independent association of selenium and *APOE* on the risk of dyslipidemia in the present study.

For the general population, the primary pathway of exposure to selenium is food, followed by water and air [60]. Selenium content in foods varies greatly depending on the selenium content of the soil where plants are grown while up to 10-fold differences in selenium contents can be found in the same food item [61]. Dietary selenium is found to be highly bioavailable and its elimination in humans was shown to be in three phases with the last phase lasting as long as 200 days [62]. Nail selenium was used as the biomarker to measure individual selenium exposure in our study for two reasons: nail samples were believed to provide an accurate measure of long-term exposure to selenium [62] and nail selenium measures are stable and do not fluctuate greatly with daily selenium intake in the diet [63]. In a previous study of the same cohort [23], we have confirmed that nail selenium levels were significantly correlated with selenium levels measured in blood, food samples and dietary intake derived from food frequency questionnaire. Positive correlations between nail selenium and selenium in urine, whole blood and serum were also confirmed in other studies [64–66]. However, since in this study hair samples were not collected, a reliable comparison between our observations and results on selenium measured in hair samples is not available.

Selenium levels in various cohorts differ by the geographic locations of the study population [67]. Although US cancer studies reported mean nail selenium levels of 0.8 *μ*g/g in control subjects, European cohorts included many control groups with nail selenium levels around 0.5 *μ*g/g, overlapping with the selenium range in our cohort. It is worth noting that the selenium levels reported in cohorts from developed countries may also be influenced by dietary supplements and, hence, may not be reflective of lifelong exposure. Recommended daily selenium intakes were established to maximize plasma glutathione peroxidases activity [68]. However, it remains to be seen whether these recommended levels are optimal for various health outcomes.

Our study has a number of strengths. The first is the relatively large sample size ensuring adequate statistical power. The second is the relatively low selenium level in the study population without selenium supplementation, which provided an opportunity to explore the associations between selenium exposure and dyslipidemia risk. The third is selenium measurements in nail samples, which provided a relatively long-term measure of exposure compared with selenium measured in blood or urine samples. For life-long rural residents consuming local food, nail selenium level closely reflects life-long selenium exposure level [62].

Our study is limited by its cross-sectional design and the results are subject to the bias of reverse causation. Longitudinal studies will be needed to confirm our results. In addition, our study result is limited to subjects older than 65 years of age. Therefore, it is not known whether the association between selenium level and the risk of dyslipidemia holds in populations of younger subjects. Furthermore, only *APOE* genotype was examined in the present study. Additional genetic and environmental confounders should be explored for the better understanding of the relationship between selenium level and dyslipidemia.

In summary, our results suggest long-term selenium higher exposure level may be associated with the risk of dyslipidemia in the elderly population. Future studies are needed to confirm our findings and to examine the underlying mechanism of the association.

## Supporting Information

S1 File Click here for additional data file. (XLS)

## References

[1] Rayman MP. The importance of selenium to human health. Lancet.2000; 356(9225):233–241. 10963212 10.1016/S0140-6736(00)02490-9

[2] Navas-Acien A, Bleys J, Guallar E. Selenium intake and cardiovascular risk: what is new? Curr Opin Lipidol.2008; 19(1): 43–49. 18196986 10.1097/MOL.0b013e3282f2b261

[3] Huang K, Liu H, Chen Z, Xu H. Role of selenium in cytoprotection against cholesterol oxide-induced vascular damage in rats. Atherosclerosis.2002; 162(1):137–144. 11947907 10.1016/s0021-9150(01)00707-9

[4] Tanguy S, Toufektsian MC, Besse S, Ducros V, De Leiris J, Boucher F. Dietary selenium intake affects cardiac susceptibility to ischaemia/reperfusion in male senescent rats. Age Ageing. 2003; 32(3):273–278. 12720612 10.1093/ageing/32.3.273

[5] Tanguy S, Morel S, Berthonneche C, Toufektsian MC, de Lorgeril M, Ducros V, et al Preischemic selenium status as a major determinant of myocardial infarct size in vivo in rats. Antioxid Redox Signal. 2004; 6(4):792–796. 15242560 10.1089/1523086041361631

[6] Ayaz M, Ozdemir S, Ugur M, Vassort G, Turan B. Effects of selenium on altered mechanical and electrical cardiac activities of diabetic rat. Arch Biochem Biophys. 2004; 426(1):83–90. 15130786 10.1016/j.abb.2004.03.030

[7] Dhingra S, Bansal M. Attenuation of LDL receptor gene expression by selenium deficiency during hypercholesterolemia. Mol Cell Biochem. 2006; 282(1–2):75–82. 16317514 10.1007/s11010-006-1266-1

[8] Flores-Mateo G, Navas-Acien A, Pastor-Barriuso R, Guallar E. Selenium and coronary heart disease: a meta-analysis. Am J Clin Nutr. 2006; 84(4):762–773. 17023702 10.1093/ajcn/84.4.762 PMC1829306

[9] Alissa EM, Bahijri SM, Ferns GA. The controversy surrounding selenium and cardiovascular disease: a review of the evidence. Med Sci Monit. 2003; 9(1):RA9–18. 12552253

[10] Venardos KM, Perkins A, Headrick J, Kaye DM. Myocardial ischemia-reperfusion injury, antioxidant enzyme systems, and selenium: a review. Curr Med Chem. 2007; 14(14):1539–1549. 17584062 10.2174/092986707780831078

[11] Mozaffarian D. Fish, mercury, selenium and cardiovascular risk: current evidence and unanswered questions. Int J Environ Res Public Health. 2009; 6(6):1894–1916. 10.3390/ijerph6061894 19578467 PMC2705224

[12] Bleys J, Miller ER 3rd, Pastor-Barriuso R, Appel LJ, Guallar E. Vitamin-mineral supplementation and the progression of atherosclerosis: a meta-analysis of randomized controlled trials. Am J Clin Nutr. 2006; 84(4):880–887, quiz 954–955. 17023716 10.1093/ajcn/84.4.880

[13] Brown KM, Arthur JR. Selenium, selenoproteins and human health: a review. Public Health Nutr. 2001; 4(2B):593–599. 11683552 10.1079/phn2001143

[14] El-Demerdash FM, Nasr HM. Antioxidant effect of selenium on lipid peroxidation, hyperlipidemia and biochemical parameters in rats exposed to diazinon. J Trace Elem Med Biol. 2014; 28(1):89–93. 10.1016/j.jtemb.2013.10.001 24188896

[15] Seale LA, Hashimoto AC, Kurokawa S, Gilman CL, Seyedali A, Bellinger FP, et al Disruption of the selenocysteine lyase-mediated selenium recycling pathway leads to metabolic syndrome in mice. Mol Cell Biol. 2012; 32(20):4141–54. 22890841 10.1128/MCB.00293-12 PMC3457337

[16] Djeffal A, Messarah M, Boumendjel A, Kadeche L, El Feki A. Protective effects of vitamin C and selenium supplementation on methomyl-induced tissue oxidative stress in adult rats. Toxicol Ind Health. 2012 Available: http://tih.sagepub.com/content/early/2012/12/05/0748233712468020.long. Accessed 6 December 2012. 10.1177/0748233712468020 23222694

[17] Rayman MP, Stranges S, Griffin BA, Pastor-Barriuso R, Guallar E. Effect of supplementation with high-selenium yeast on plasma lipids: a randomized trial. Ann Intern Med. 2011; 154(10): 656–665. 10.7326/0003-4819-154-10-201105170-00005 21576533

[18] Bleys J, Navas-Acien A, Stranges S, Menke A, Miller ER 3rd, Guallar E.Serum selenium and serum lipids in US adults. Am J Clin Nutr. 2008; 88(2):416–423. 18689378 10.1093/ajcn/88.2.416 PMC2553708

[19] Laclaustra M, Stranges S, Navas-Acien A, Ordovas JM, Guallar E. Serum selenium and plasma lipids in US adults: National Health and Nutrition Examination Survey (NHANES) 2003–2004. Atherosclerosis. 2010; 210(2):643–648. 10.1016/j.atherosclerosis.2010.01.005 20102763 PMC2878899

[20] Stranges S, Laclaustra M, Ji C, Cappuccio FP, Navas-Acien A, Ordovas JM, et al Higher selenium status is associated with adverse blood lipid profile in British adults. J Nutr. 2010; 140(1):81–87. 10.3945/jn.109.111252 19906812 PMC2793123

[21] Fülöp P, Seres I, Jenei Z, Juhász I, Paragh G. Increased hair selenium concentration in hyperlipidemic patients. J Cell Mol Med. 2013; 17(3):350–5. 10.1111/jcmm.12013 23402643 PMC3823016

[22] Stranges S, Tabák AG, Guallar E, Rayman MP, Akbaraly TN, Laclaustra M, et al Selenium status and blood lipids: the cardiovascular risk in young finns study. J Intern Med. 2011; 270(5):469–477. 10.1111/j.1365-2796.2011.02398.x 21554435 PMC3172343

[23] Gao S, Jin Y, Hall KS, Liang C, Unverzagt FW, Ji R, et al Selenium level and cognitive function in rural elderly Chinese. Am J Epidemiol. 2007; 165(8):955–965. 17272290 10.1093/aje/kwk073 PMC2760949

[24] Su L, Cheng Y, Liang C, Ma F, Bian J, Li P, et al Preliminary study on the relationship between low environmental selenium exposure and serum lipids level among the elderly rural chinese. Journal of Environmental Hygiene. 2013; 3(2): 84–87.

[25] Gao S, Jin Y, Hall KS, Liang C, Unverzagt FW, Ma F, et al Selenium level is associated with apoE epsilon4 in rural elderly Chinese. Public Health Nutr. 2009; 12(12):2371–2376. 10.1017/S1368980009005102 19278567 PMC2842076

[26] Cao J, Liu Y, Shen J, Li P, Gao S. Study on environmental selenium level of four areas in Shandong and Sichuan province. Chinese Journal of Preventive Medicine. 2007; 41(5):419–421.18206021

[27] Joint committee for developing Chinese guidelines on prevention and treatment of dyslipidemia in adults. Chinese guidelines on prevention and treatment of dyslipidemia in adults. Chinese Journal of Cardiology. 2007; 35(5):390–419. 17711682

[28] Kim SW, Heo JH, Kim CH, Yoo DC, Won DH, Lee SG, et al Rapid and direct detection of apolipoprotein E genotypes using whole blood from humans. J Toxicol Environ Health A. 2010; 73(21–22):1502–1510. 10.1080/15287394.2010.511573 20954076

[29] IPAQ group. Guidelines for data processing and analysis of theInternational Physical Activity Questionnaire (IPAQ). 2005 Available: http://www.ipaq.ki.se/scoring.html.

[30] Obeid O, Elfakhani M, Hlais S, Iskandar M, Batal M, Mouneimne Y, et al Plasma Copper, Zinc, and Selenium Levels and Correlates with Metabolic Syndrome Components of Lebanese Adults. Biol Trace Elem Res. 2008; 123(1–3):58–65. 10.1007/s12011-008-8112-0 18288450

[31] Suadicani P, Hein HO, Gyntelberg F. Serum selenium concentration and risk of ischaemic heart disease in a prospective cohort study of 3000 males. Atherosclerosis.1992; 96(1):33–42. 1418100 10.1016/0021-9150(92)90035-f

[32] Coudray C, Roussel AM, Mainard F, Arnaud J, Favier A. Lipid peroxidation level and antioxidant micronutrient status in a pre-aging population; correlation with chronic disease prevalence in a French epidemiological study (Nantes, France). J Am Coll Nutr. 1997; 16(6):584–591. 9430087

[33] Jossa F, Trevisan M, Krogh V, Farinaro E, Giumetti D, Fusco G, et al Serum selenium and coronary heart disease risk factors in southern Italian men. Atherosclerosis. 1991; 87(2–3):129–134. 1854360 10.1016/0021-9150(91)90015-u

[34] Bates CJ, Thane CW, Prentice A, Delves HT. Selenium status and its correlates in a British national diet and nutrition survey: people aged 65 years and over. J Trace Elem Med Biol. 2002; 16(1):1–8. 11878747 10.1016/s0946-672x(02)80002-5

[35] Salonen JT, Salonen R, Seppänen K, Kantola M, Parviainen M, Alfthan G, et al Relationship of serum selenium and antioxidants to plasma lipoproteins, platelet aggregability and prevalent ischaemic heart disease in Eastern Finnish men. Atherosclerosis. 1988; 70(1–2):155–160. 3258519 10.1016/0021-9150(88)90109-8

[36] Karita K, Yamanouchi Y, Takano T, Oku J, Kisaki T, Yano E. Associations of blood selenium and serum lipid levels in Japanese premenopausal and postmenopausal women. Menopause. 2008; 15(1):119–124. 18257145 10.1097/gme.0b013e31806bf32c

[37] Moosmann B, Behl C.Selenoprotein synthesis and side-effects of statins.Lancet. 2004;363:892–894. 15031036 10.1016/S0140-6736(04)15739-5

[38] Arnaud J, Akbaraly TN, Hininger-Favier I, Berr C, Roussel AM.Fibrates but not statins increase plasma selenium in dyslipidemic aged patients-the EVA study. J Trace Elem Med Biol. 2009; 23:21–28. 10.1016/j.jtemb.2008.08.001 19203713

[39] Stranges S, Marshall JR, Trevisan M, Natarajan R, Donahue RP, Combs GF, et al Effects of selenium supplementation on cardiovascular disease incidence and mortality: secondary analyses in a randomized clinical trial. American Journal of Epidemiology. 2006; 163(8):694–699. 16495471 10.1093/aje/kwj097

[40] Klein EA, Thompson IM Jr, Tangen CM, Crowley JJ, Lucia MS, Goodman PJ, et al Vitamin E and the risk of prostate cancer: the Selenium and Vitamin E Cancer Prevention Trial (SELECT). JAMA. 2011;306(14):1549–1556. 10.1001/jama.2011.1437 21990298 PMC4169010

[41] Lippman SM, Klein EA, Goodman PJ, Lucia MS, Thompson IM, Ford LG, et al Effect of selenium and vitamin E on risk of prostate cancer and other cancers. The Selenium and Vitamin E Cancer Prevention Trial (SELECT). JAMA. 2009; 301(1):39–51. 10.1001/jama.2008.864 19066370 PMC3682779

[42] Galan-Chilet I, Tellez-Plaza M, Guallar E, De Marco G, Lopez-Izquierdo R, Gonzalez-Manzano I, et al Plasma selenium levels and oxidative stress biomarkers: A gene-environment interaction population-based study. Free Radic Biol Med. 2014; 74:229–236. 10.1016/j.freeradbiomed.2014.07.005 25017966

[43] Ducros V, Laporte F, Belin N, David A, Favier A. Selenium determination in human plasma lipoprotein fractions by mass spectrometry analysis. J Inorg Biochem. 2000; 15;81(1–2):105–109. 11001438 10.1016/s0162-0134(00)00071-4

[44] Olson GE, Winfrey VP, Hill KE, Burk RF. Megalin mediates selenoprotein P uptake by kidney proximal tubule epithelial cells. J Biol Chem. 2008; 283:6854–6860. 10.1074/jbc.M709945200 18174160

[45] Stranges S, Navas-Acien A, Rayman MP, Guallar E. Selenium status and cardiometabolic health: state of the evidence. Nutr Metab Cardiovasc Dis. 2010; 20(10):754–760. 10.1016/j.numecd.2010.10.001 21094028

[46] Végh C, Langmár Z, Szerző M, Agota A, Marosi K, Szabolcs Z, et al Connections between apolipoprotein E genotypes and the development of cardiovascular diseases. Orv Hetil. 2012; 153(52):2070–2076. 10.1556/OH.2012.29508 23261995

[47] Kofler BM, Miles EA, Curtis P, Armah CK, Tricon S, Grew J, et al Apolipoprotein E genotype and the cardiovascular disease risk phenotype: impact of sex and adiposity (the FINGEN study). Atherosclerosis. 2012; 221:467–470. 10.1016/j.atherosclerosis.2012.01.042 22365656

[48] Kotaska K, Kolarova J, Kotrcova K, Cepova J, Prusa R. Correlation between common genetic variants and risk factors associated with prediction of cardiovascular diseases in dyslipidemic patients. Genet Test Mol Biomarkers. 2012; 16(3):210–214. 10.1089/gtmb.2011.0129 21919778

[49] Ward H, Mitrou PN, Bowman R, Luben R, Wareham NJ, Khaw KT, et al APOE genotype, lipids, and coronary heart disease risk: a prospective population study. Arch Intern Med. 2009; 169(15):1424–1429. 10.1001/archinternmed.2009.234 19667307

[50] Hu M, Mak VW, Tomlinson B. Polymorphisms in apolipoprotein E and apolipoprotein A-V do not influence the lipid response to rosuvastatin but are associated with baseline lipid levels in Chinese patients with hyperlipidemia. J Clin Lipidol. 2012; 6(6):585–592. 10.1016/j.jacl.2012.02.005 23312054

[51] Yilmaz-Aydogan H, Kurnaz O, Kucukhuseyin O, Akadam-Teker B, Kurt O, Eronat AP, et al Different effects of PPARA, PPARG and ApoE SNPs on serum lipids in patients with coronary heart disease based on the presence of diabetes. Gene. 2013; 523(1):20–26. 10.1016/j.gene.2013.03.136 23583468

[52] Smalinskiene A, Petkeviciene J, Luksiene D, Jureniene K, Klumbiene J, Lesauskaite V. Association between APOE, SCARB1, PPARα polymorphisms and serum lipids in a population of Lithuanian adults. Lipids Health Dis. 2013; 12:120 10.1186/1476-511X-12-120 23919842 PMC3751123

[53] Petkeviciene J, Smalinskiene A, Luksiene DI, Jureniene K, Ramazauskiene V, Klumbiene J, et al Associations between Apolipoprotein E Genotype, Diet, Body Mass Index, and Serum Lipids in Lithuanian Adult Population. PLoS One. 2012; 7(7):e41525 10.1371/journal.pone.0041525 22844488 PMC3402524

[54] Dhingra S, Bansal M. Modulation of hypercholesterolemia-induced alterations in apolipoprotein B and HMG-CoA reductase expression by selenium supplementation. Chem Biol Interact. 2006; 161(1):49–56. 16581047 10.1016/j.cbi.2006.02.008

[55] Henriques AD, Tonet-Furioso AC, Machado-Silva W, Freitas WM, Quaglia LA, Santos SN, et al Apoliprotein E genotype is associated with apoliprotein B plasma levels but not with coronary calcium score in very elderly individuals in primary care setting. Gene. 2014; 539(2):275–278. 10.1016/j.gene.2014.01.077 24530308

[56] Dhingra S, Bansal M. Attenuation of LDL receptor gene expression by selenium deficiency during hypercholesterolemia. Mol Cell Biochem. 2006; 282(1–2):75–82. 16317514 10.1007/s11010-006-1266-1

[57] Dhingra S, Bansal M. Hypercholesterolemia and LDL receptor mRNA expression: modulation by selenium supplementation. Biometals. 2006; 19(5):493–501. 16937255 10.1007/s10534-005-5393-z

[58] Sengupta A, Carlson BA, Hoffmann VJ, Gladyshev VN, Hatfield DL. Loss of housekeeping selenoprotein expression in mouse liver modulates lipoprotein metabolism. Biochem Biophys Res Commun. 2008; 365(3):446–452. 17996733 10.1016/j.bbrc.2007.10.189 PMC2175387

[59] Hubacek JA, Vrablik M. Effect of apolipoprotein E polymorphism on statin-induced decreases in plasma lipids and cardiovascular events. Drug Metabol Drug Interact.2011;26(1):13–20. 10.1515/DMDI.2011.107 21557673

[60] Barceloux DG. Selenium.J Toxicol Clin Toxicol. 1999;37(2):145–172. 10382553 10.1081/clt-100102417

[61] World Health Organization. Selenium Environmental Health Criteria 58: A report of the International Programme on Chemical Safety. World Health Organization, Geneva, 1987.

[62] He K. Trace elements in nails as biomarkers in clinical research.Eur J Clin Invest.2011; 41(1):98–102. 10.1111/j.1365-2362.2010.02373.x 20813017 PMC2998551

[63] Krogh V, Pala V, Vinceti M, Berrino F, Ganzi A, Micheli A, et al Toenail selenium as biomarker: reproducibility over a one-year period and factors influencing reproducibility. J Trace Elem Med Biol.2003; 17 Suppl 1:31–36. 14650626

[64] Ji A, Guo Y, Feng Z, Yang Q, Wang D, Yang W. Measurement of selenium content of nail and hair and correlation studies for Kaschin-Beck patients. Chinese Journal of Control of Endemic Disease.1991; 6(6):327–328.

[65] Slotnick MJ, Nriagu JO. Validity of human nails as a biomarker of arsenic and selenium exposure: A review. Environ Res.2006; 102(1):125–139. 16442520 10.1016/j.envres.2005.12.001

[66] Ovaskainen ML, Virtamo J, Alfthan G, Haukka J, Pietinen P, Taylor PR, et al Toenail selenium as an indicator of selenium intake among middle-aged men in an area with low soil selenium. Am J Clin Nutr.1993; 57(5): 662–665. 8480683 10.1093/ajcn/57.5.662

[67] Zhuo H, Smith AH, Steinmaus C. Selenium and lung cancer:a quantitative analysis of heterogeneity in the current epidemiological literature. Cancer Epidemiol Biomarkers Prev. 2004;13(5):771–778. 15159309

[68] Thomson CD. Assessment of requirements for selenium and adequacy of selenium status: a review. Eur J Clin Nutr. 2004; 58(3):391–402. 14985676 10.1038/sj.ejcn.1601800

